# Persistence of Activated and Adaptive-Like NK Cells in HIV^+^ Individuals despite 2 Years of Suppressive Combination Antiretroviral Therapy

**DOI:** 10.3389/fimmu.2017.00731

**Published:** 2017-06-30

**Authors:** Anna C. Hearps, Paul A. Agius, Jingling Zhou, Samantha Brunt, Mkunde Chachage, Thomas A. Angelovich, Paul U. Cameron, Michelle Giles, Patricia Price, Julian Elliott, Anthony Jaworowski

**Affiliations:** ^1^Centre for Biomedical Research, Burnet Institute, Melbourne, VIC, Australia; ^2^Department of Infectious Diseases, Monash University, Melbourne, VIC, Australia; ^3^Department of Epidemiology and Preventive Medicine, Monash University, Melbourne, VIC, Australia; ^4^Centre for Population Health, Burnet Institute, Melbourne, VIC, Australia; ^5^Pathology and Laboratory Medicine, University of Western Australia, Perth, WA, Australia; ^6^Department of Microbiology and Immunology, Doherty Institute for Infection and Immunity, The University of Melbourne, Melbourne, VIC, Australia; ^7^Infectious Diseases Unit, Alfred Hospital, Melbourne, VIC, Australia; ^8^School of Biomedical Science, Curtin University, Perth, WA, Australia

**Keywords:** NK cell, HIV, adaptive-like NK cell, immune activation, combination antiretroviral therapy

## Abstract

Innate immune dysfunction persists in HIV^+^ individuals despite effective combination antiretroviral therapy (cART). We recently demonstrated that an adaptive-like CD56^dim^ NK cell population lacking the signal transducing protein FcRγ is expanded in HIV^+^ individuals. Here, we analyzed a cohort of HIV^+^ men who have sex with men (MSM, *n* = 20) at baseline and following 6, 12, and 24 months of cART and compared them with uninfected MSM (*n* = 15) to investigate the impact of cART on NK cell dysfunction. Proportions of NK cells expressing markers of early (CD69^+^) and late (HLA-DR^+^/CD38^+^) activation were elevated in cART-naïve HIV^+^ MSM (*p* = 0.004 and 0.015, respectively), as were FcRγ^−^ NK cells (*p* = 0.003). Using latent growth curve modeling, we show that cART did not reduce levels of FcRγ^−^ NK cells (*p* = 0.115) or activated HLA-DR^+^/CD38^+^ NK cells (*p* = 0.129) but did reduce T cell and monocyte activation (*p* < 0.001 for all). Proportions of FcRγ^−^ NK cells were not associated with NK cell, T cell, or monocyte activation, suggesting different factors drive CD56^dim^ FcRγ^−^ NK cell expansion and immune activation in HIV^+^ individuals. While proportions of activated CD69^+^ NK cells declined significantly on cART (*p* = 0.003), the rate was significantly slower than the decline of T cell and monocyte activation, indicating a reduced potency of cART against NK cell activation. Our findings indicate that 2 years of suppressive cART have no impact on CD56^dim^ FcRγ^−^ NK cell expansion and that NK cell activation persists after normalization of other immune parameters. This may have implications for the development of malignancies and co-morbidities in HIV^+^ individuals on cART.

## Introduction

Effective combination antiretroviral therapy (cART) suppresses HIV replication and prevents AIDS-related illness but does not eliminate HIV or fully restore immune function. Virologically suppressed HIV^+^ individuals show phenotypic and functional evidence of persistent immune dysfunction, particularly within the innate immune system (1). We and others have demonstrated that heightened HIV-related activation of NK cells (2, 3) and monocytes (4–8) persist in cART-treated individuals despite undetectable levels of HIV viremia (<20 HIV RNA copies/mL plasma). Markers of innate immune activation/inflammation are associated with co-morbidities such as cardiovascular disease, neurocognitive impairment, and malignancies in HIV^+^ individuals [reviewed in Ref. (9)] and also predict mortality in this population (10). This suggests persistent innate immune activation may have a detrimental effect on the long-term health of HIV^+^ individuals on cART. Elucidating the underlying mechanism of this effect is essential for preserving the health of the estimated 17 million HIV^+^ individuals worldwide currently receiving cART.

Acute HIV infection triggers a short-lived expansion of the mature CD56^dim^CD16^+^ NK cell subset (which declines during progressive infection) and the emergence of a population of functionally anergic CD56^−^ NK cells (11–13), but these defects are largely reversed by cART (14). In contrast, our cross-sectional study demonstrated increased NK cell activation and heightened spontaneous degranulation in both viremic and virologically suppressed HIV^+^ individuals (2), indicating NK cell activation persists despite effective cART. However, the duration of this effect and its impact on co-morbid disease remain unknown.

Although considered innate immune cells, increasing evidence indicates NK cells also possess adaptive, memory-like properties similar to cytotoxic CD8^+^ T cells (15). A rapid expansion of NK cells able to target murine cytomegalovirus (MCMV)-infected cells has been demonstrated following primary MCMV infection (16). Furthermore, NK cells exhibiting memory-like properties persisted in tissue for several months after MCMV infection and displayed rapid degranulation upon subsequent stimulation. A similar expansion and persistence of specific populations of NK cells also occurs in response to human CMV (HCMV) infection; HCMV seropositivity is associated with expansion of CD56^dim^ NK cells expressing the activating receptor NKG2C and a pattern of killer cell immunoglobulin-like receptors consistent with clonal expansion (17–19). Recent studies associate HCMV infection with expansion of multiple subsets of adaptive-like NK cells, including (but not limited to) those expressing NKG2C (20–22). These “imprinted” populations are stably maintained for at least 15 months following infection (23) and show enhanced antibody-dependent activation (21), consistent with an important role in protective immunity against viral infections. Clonal expansion of NK cells is also observed early after Chikungunya (24) and Hantavirus (25) infections; and we have previously reported expansion of adaptive-like FcRγ^−^ NK cells in HIV infection (3); however, it remains unclear whether pre-existing CMV infection is a prerequisite for the observed expansion of specific NK cell populations in these settings.

NK cell receptor profiles are perturbed in viremic HIV^+^ individuals, with increased expression of the activating receptor NKG2C and reduced expression of the inhibitory receptor NKG2A on NK cells (26, 27). The proportion of NKG2A^+^ NK cells is restored in cART-treated HIV^+^ individuals in some (27, 28) but not all studies (26); however, expansion of NKG2C^+^ NK cells can persist in aviremic individuals (29) despite at least 2 years of viral suppression (27). HIV-associated expansion of NKG2C^+^ NK cells appears to occur only in individuals seropositive for HCMV (27, 29), and HCMV seropositivity is also a prerequisite for NKG2C^+^ NK cell expansion induced by other chronic viral infections such as hepatitis B and C infection (18). These findings suggest chronic viral diseases such as HIV and HCMV may act synergistically to heighten immune dysfunction. Accordingly, HCMV^+^/HIV^+^ individuals with >12 years of successful cART have higher levels of HCMV-reactive antibodies and T cells than HCMV^+^/HIV^−^ individuals (30, 31). These findings highlight the necessity to consider HCMV antibodies in studies of NK cell dysfunction and underscore the requirement for appropriate HIV^−^ comparison groups when analyzing these defects in HIV^+^ populations who carry a higher burden of HCMV than the general population.

In a recent cross-sectional study, we made the novel discovery that a population of CD56^dim^ NK cells lacking the intracellular signal transduction protein FcRγ is expanded in both viremic and virologically suppressed HIV^+^ individuals (3). FcRγ is an immunoreceptor tyrosine-based activation motif-containing adaptor protein responsible for transducing signals through activating NK cell receptors such as CD16, and acting as a chaperone for these receptors. These CD56^dim^FcRγ^−^ NK cells have reduced expression of CD16 and the natural cytotoxicity receptors NKp30 and NKp46, but enhanced antibody-dependent cell-mediated cytotoxicity (ADCC) activity, and represent up to 90% of the NK cell population in some HIV^+^ individuals (3). An analogous population of CD56^dim^FcRγ^−^ NK cells has previously been characterized in HIV^−^/HCMV^+^ individuals and shown to possess a memory-like phenotype with adaptive immune features including enhanced ADCC against target cells infected with HCMV or herpes simplex virus, implying a specialized role in antibody-dependent cross-protection (22, 32). We therefore investigated the HIV-related expansion of adaptive-like CD56^dim^FcRγ^−^ NK cells in a contemporary cohort of HIV^+^ men who have sex with men (MSM). Given the abovementioned effect of HCMV infection on NK cell imprinting and the near ubiquitous HCMV seropositivity of HIV^+^ MSM, we used a novel longitudinal MSM cohort to study the effect of HIV and cART on the prevalence of FcRγ^−^ NK cells by comparison with appropriately matched HIV^−^ MSM. Furthermore, we used latent growth curve modeling to quantify the rate at which NK cell activation is reversed following viral suppression as compared to activation of other immune cell compartments.

## Materials and Methods

### Study Participants

Participants were identified from the Melbourne HIV Cohort, a prospective study of HIV-positive and HIV-seronegative men who self-report having sex with men. Participants in the Melbourne HIV Cohort were reviewed annually to assess co-morbidities. Peripheral blood mononuclear cells (PBMC) and plasma were prepared and archived from each visit. Baseline samples were analyzed from 20 cART-naïve HIV^+^ MSM and 15 HIV^−^ MSM matched for age with the HIV^+^ MSM at the baseline time-point. HIV^+^ individuals were recruited when they were cART-naïve and followed-up every 3 months for 12 months following cART-initiation, then annually thereafter. Of the 20 HIV^+^ MSM, one initiated a cART regimen consisting of efavirenz, festinavir and lamivudine. The other 19 participants received a cART regimen of tenofovir and emtricitabine, plus either efavirenz (*n* = 6), rilpivirine (*n* = 5), raltegravir (*n* = 3), ritonavir + atazanavir (*n* = 4), or ritonavir + neviripine (*n* = 1); two individuals had their regimen altered (from raltegravir to dolutegravir and from efavirenz to raltegravir) during the follow up period. At the time of the study, 10 of the HIV^+^ MSM had reached the 24-month post-cART initiation time-point and were included in the analysis. Exclusion criteria included co-morbid disease (e.g., cardiovascular disease, diabetes) and current use of statins, steroids, or other anti-inflammatory medications. For selected experiments, an additional 14 HIV^−^ men of a similar age were recruited from the general community. Ethical approval for this study was obtained from the Alfred Hospital Research and Ethics Committee.

### Sample Processing and Immunophenotyping

Cells and plasma were prepared from whole blood collected into acid citrate dextrose tubes. PBMC were collected following Ficoll density gradient centrifugation of blood and stored in liquid N_2_. Cells were stained with LIVE/DEAD^®^ fixable dead cell stain (ThermoFisher Scientific, Waltham, MA, USA) prior to immunophenotyping. Expression of surface receptors on NK cells, monocytes, and T cells were detected by staining with the following antibodies: CD56 APC (clone NKH-1) from Beckman Coulter (Brea, CA); CD14-V500 (clone M5E2), CD16 PE-Cy7 (clone 3G8), CD3 PerCP-Cy5.5 (clone UCHT1), CD38-PE (clone HB7), HLA-DR FITC or APC-H7 (clone G46-6), CD4 PE-Cy7 (clone RPA-TA), CD8 APC-H7 (clone SK1), all from BD Biosciences (San Jose, CA, USA); CD3 BV510 (clone OKT3), CD56 AF700 (clone HCD 56), CD57 Pacific Blue (clone HCD57), CD355 PerCP-Cy5.5 (NKp46, clone 9E2), CD337 AlexaFluor 647 (NKp30, clone CD337), all from Biolegend (San Diego, CA, USA). Expression of intracellular FcRγ was detected after labeling of surface antigens and following permeabilization with Perm/Wash buffer 1 (BD Biosciences), then staining with anti-FcRγ FITC (FcεR1, γ subunit, rabbit polyclonal, Millipore, Darmstadt, Germany). The specificity of the polyclonal anti-FcRγ FITC antibody has been previously demonstrated (3). Cells were acquired on a Fortessa LSR flow cytometer (BD Biosciences) and data analyzed using FlowJo software (version 10, FlowJo LLC, Ashland, OR, USA). Gating strategies for each cell type are depicted in Figure S1 in Supplementary Material.

### Measurement of Plasma Inflammatory Markers and HCMV Antibody

Plasma concentrations of soluble CD163 (sCD163) and CXCL10 were measured using commercial ELISA kits as per manufacturer’s instructions (Macro 163, IQ Products, Groningen, Netherlands and DIP-100, Quantikine ELISA, R&D Systems, Minneapolis, MN, USA, respectively). IgG reactive with HCMV was quantified using HCMV lysate, HCMV glycoprotein B (gB), and HCMV IE-1 antigens as previously described (3). CMV seropositivity was defined as >2 SD above the mean antibody levels for HCMV lysate derived for a set of 11 samples that had been deemed seronegative by the ARCHITECT CMV IgG assay (Abbott Diagnostics, IL). Data are presented in arbitrary units (AU) defined relative to a standard plasma pool run on each plate. Cross-reactivity of the CMV ELISA with other herpes viruses has not been formally assessed. Samples were measured over a range of dilutions to ensure accurate quantitation in the high range.

### Statistical Analyses

Cross-sectional comparisons of marker levels between groups were made using Mann–Whitney *U*-test, while differences between baseline and post-cART time points in HIV^+^ MSM were made using Wilcoxon matched pairs signed rank test (GraphPad Prism software, version 6.05). Multilevel modeling was used to estimate latent growth-curve models exploring the subject-specific nature of the association between each marker and time. Latent growth-curve models were also estimated on the natural log of each immunological marker and post-estimation non-linear equations using exponentiated model coefficients were estimated to provide proportional rates of immunological change at specific time-points. Latent growth-curve models comprised two-levels, HIV^+^ individuals at level-2 (i.e., random intercept and coefficient for time) and their marker responses over-time at level-1 (see Supplementary Eq. 1 in Supplementary Material). Latent growth-curve modeling was extended to incorporate more complex bivariate outcome models (i.e., two outcomes), enabling simultaneous estimation of log-marker rates of change and post-estimation inference comparing rates of change between different markers (i.e., modeling the comparative mean per-cent change between two markers over-time, and accounting for the correlation between individuals’ responses to these two markers across time). In these models, typically, random effects for heterogeneity in both participants’ baseline marker levels (i.e., random intercept) and the nature of any change in marker level over time (i.e., random coefficient/slope) were estimated, with an unstructured covariance estimated between each random effect. A limitation of these analyses was that for markers measured using a binomial statistical understanding (i.e., outcomes where cell proportions were determined) the distributional assumptions of the linear mixed models used in longitudinal modeling were not entirely met. Nested model-based likelihood-ratio statistics were used to provide statistical inference for model fit when relaxing model constraints (random effects and the functional form of fixed effects for time). To assess the fit of the estimated latent growth-curve models, diagnostic plots comparing participants observed marker levels with Bayesian model-based (best linear unbiased predictions) predicted levels over time were produced and inspected. Contemporaneous (i.e., both outcome and factor variable responses from the same time-period were regressed) unadjusted longitudinal associations between selected factors and participant CD56^dim^ FcRγ^−^ NK cell proportions were estimated using multilevel modeling. In these multilevel models, factors were estimated as time-varying fixed effects with a random intercept (level-2) to account for the dependency in the data given an individual’s repeated marker level measurement over-time. Statistical inference was assessed at the 5% level. Stata version 13.1 statistical package (StataCorp LP, College Station, TX, USA) was used for multilevel modeling and bivariate latent growth-curve modeling was undertaken using the user-written Stata program generalized linear latent mixed modeling (gllamm) [(33)].

## Results

### MSM Show Expansion of FcRγ^−^ NK Cells and Elevated Plasma Levels of HCMV Antibodies as Compared to Non-MSM Individuals

**Figure 1:**
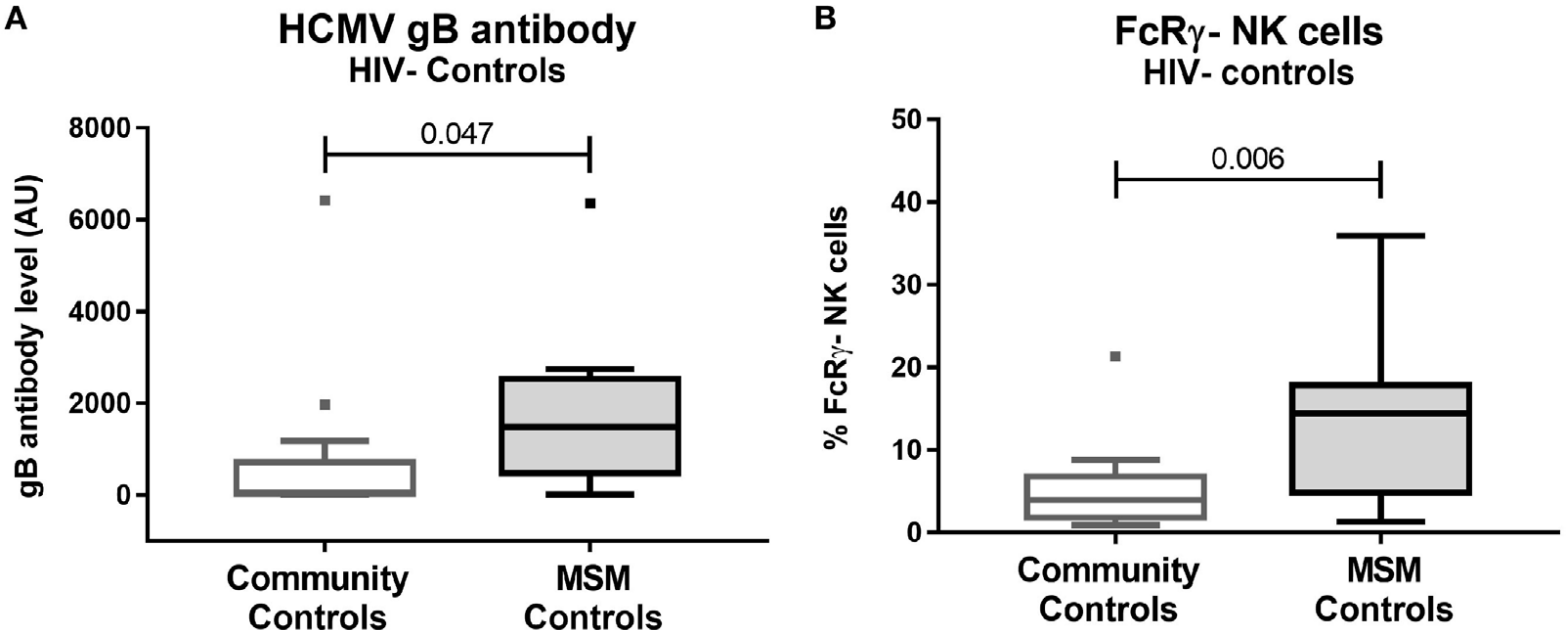
CD56^dim^ FcRγ^−^ NK cell expansion and increased human CMV (HCMV) antibodies are associated with MSM-related factors. **(A)** Plasma antibodies to the HCMV glycoprotein B (gB) were quantified by ELISA in plasma from HIV^−^ male controls recruited from either the community (*n* = 14) or from the Melbourne HIV Cohort consisting of men who have sex with men (MSM, *n* = 14). **(B)** The proportion of CD56^dim^CD16^+^ NK cells lacking the FcRγ^−^ signal transduction protein was measured in peripheral blood mononuclear cells from the same individuals as in **(A)** using intracellular staining and flow cytometry. Graphs show Tukey plots of median (bar), IQR (box), and 1.5x IQR (whiskers); outliers are indicated by squares. *p* values shown were determined by Mann–Whitney *U* test. AU, Arbitrary units.

In many developed countries, the HIV epidemic is concentrated in at-risk populations such as MSM, yet demographic and clinical differences that exist between these populations and the general community [i.e., prevalence of smoking, sexually transmitted infections, HCMV seropositivity (34)] are rarely considered in immunological studies. Given the association between HCMV infection and adaptive-like NK cell expansion in HIV seronegative individuals shown by ourselves and others (3, 22), we first asked whether proportions of FcRγ^−^ NK cells were influenced by MSM status. We compared samples from 14 non-MSM males (median age [IQR] 31.0 [30.0–39.0] years, 78.6% or 11/14 CMV-seropositive), and 14 MSM of a comparable age (35.0 [29.3–43.5] years, 85.7% or 12/14 CMV-seropositive) who were all HIV seronegative. MSM had higher levels of IgG antibodies reactive with the HCMV envelope protein gB (Figure 1A, *p* = 0.047) but also showed significantly increased proportions of FcRγ^−^ NK cells (median [IQR] 14.4% [4.8–17.8] for MSM vs 4.0% [1.8–6.8] for non-MSM; *p* = 0.006, Figure 1B). These differences persisted when only CMV seropositive individuals were compared (*p* = 0.012 and 0.037 for gB antibodies and FcRγ^−^ NK cells, respectively, not shown). These findings confirmed the importance of controlling for MSM sexual exposure and HCMV burden in our subsequent analyses of NK cell immunology in HIV infection.

### Viremic HIV Infection in MSM Is Associated with a Further Expansion of Adaptive-Like FcRγ^−^ NK Cells with a Similar Phenotype to Those in HIV-Seronegative MSM

**Figure 2:**
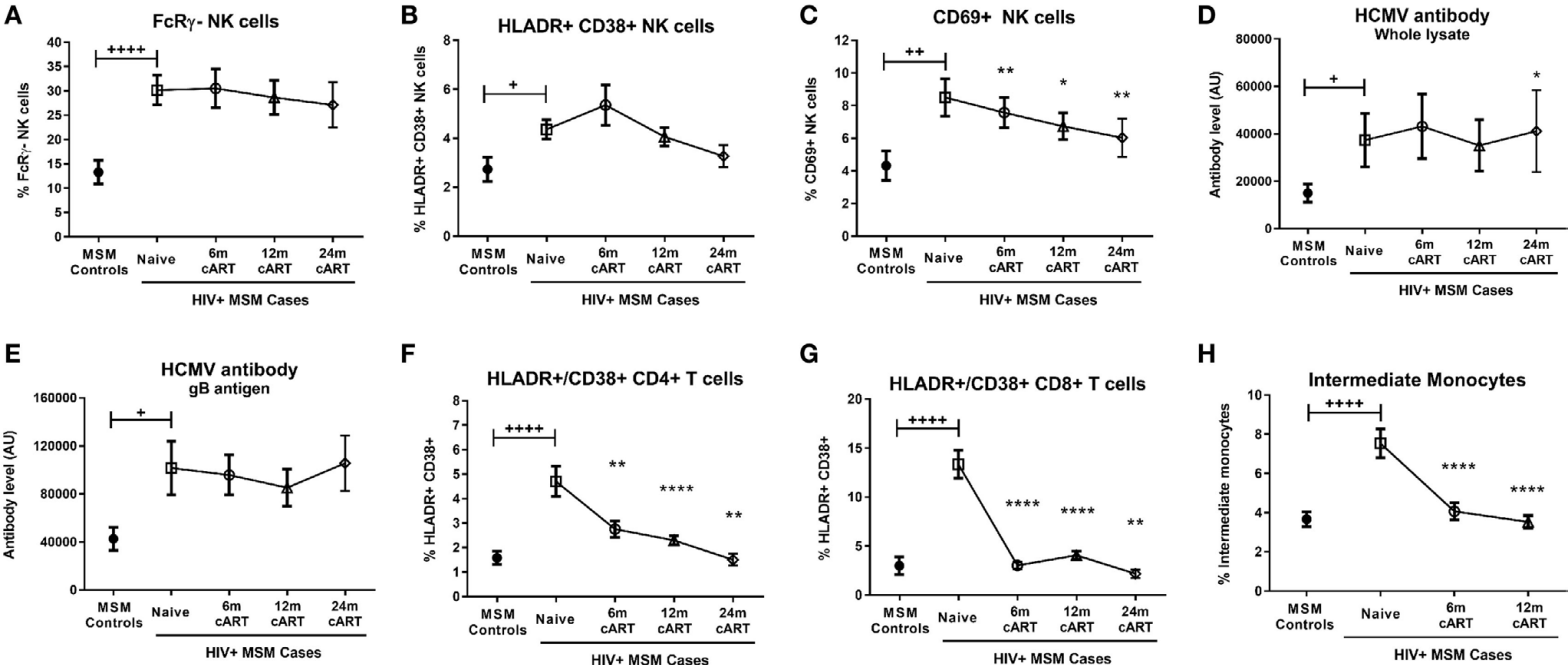
Combination antiretroviral therapy (cART) reverses T cell and monocyte activation, but not NK cell dysfunction or human CMV (HCMV) antibody levels. The proportion of FcRγ^−^ NK cells (**A**), activated HLA-DR^+^CD38^+^ **(B)**, and CD69^+^ **(C)** NK cells, plasma levels of HCMV-specific antibodies to either whole HCMV lysate **(D)** or gB antigen **(E)**, the percentage of activated HLA-DR^+^CD38^+^ CD4^+^ **(F)** and CD8^+^ **(G)** T cells and the proportion of intermediate (CD14^++^CD16^+^) monocytes **(H)** was determined in HIV-uninfected MSM (*n* = 15) and HIV^+^ MSM (*n* = 20) at baseline (cART-naïve) and after 6, 12, and 24 months of cART. Graphs show median and IQR. +, ++, and ++++ denote *p* < 0.05, 0.01, and 0.0001, respectively, as compared to HIV^−^ MSM determined by Mann–Whitney *U* test. *, **, and **** and denote *p* < 0.05, <0.01, and <0.0001, respectively, as compared to the corresponding cART-naïve value determined by Wilcoxon matched pairs signed rank test. AU, arbitrary units.

To assess the impact of untreated HIV infection on FcRγ^−^ NK cell expansion in an appropriately controlled study population, we analyzed baseline samples from cART-naïve HIV^+^ and HIV^−^ MSM of similar age (*n* = 20 and 15, respectively) from the Melbourne HIV cohort. Demographic characteristics and relevant clinical parameters are detailed in Table 1. Proportions of FcRγ^−^ NK cells were expanded in HIV^+^ MSM, with a median of 28.6% (IQR: 24.6–38.3%) compared to 14.4% (4.8–17.8%) in HIV^−^ MSM (*p* < 0.0001, Figure 2A). The phenotype of these cells in the two groups was similar; CD56^dim^ FcRγ^−^ NK cells had very low expression of the natural cytotoxicity receptors NKp30 and NKp46 and heightened expression of the maturation/differentiation marker CD57, which was statistically significant in HIV^+^ individuals; however, there was substantial inter-individual variation in the pattern of CD57 expression on FcRγ^−^ and FcRγ^+^ NK cells (Figure S2A–C in Supplementary Material). To further explore the phenotype of these cells and investigate whether they were the result of proliferative expansion, we analyzed expression of the chemokine receptor CXCR6 [indicative of tissue-homing NK cells (35)], and a proliferation marker (Ki-67) on FcRγ^−^ and FcRγ^+^ CD56^dim^ NK cells in a subset of HIV^+^ donors. Compared to FcRγ^+^ CD56^dim^ NK cells, FcRγ^−^ cells had significantly lower expression of CXCR6 (0.7 vs 5.8%; *p* = 0.004, data not shown) while both populations showed equal, low expression of Ki-67 (0.5 vs 1.0%, *p* = 0.945, data not shown). These data indicate that the proportion of adaptive-like FcRγ^−^ NK cells is expanded by HIV infection (in addition to the effects of MSM-related factors) and that this cell population does not appear to be the result of heightened proliferation or show increased expression of tissue-homing receptors but may represent a more mature NK cell subset.

**Table 1:** Demographic and clinical characteristics of HIV^−^ and HIV^+^ MSM at baseline and longitudinally post-combination antiretroviral therapy (post-cART) initiation.^a^

	HIV^−^ MSM	HIV^+^ MSM			
			Post-cART follow-up time point		
Median (IQR)	Baseline	Baseline	6 months	12 months	24 months
*n*	15	20	20	20	10
Age (years)	34.0 (29.0–43.0)	32.0 (29.0–43.5)			
Current smoker, *n* (%)	1 (6.6%)	4 (20%)			
HCV^+^ (antigen and PCR^+^), *n* (%)	0	2 (10%)			
Human CMV seropositive	13 (86.7%)	20 (100%)^b^			
Nadir CD4 T cell count (cells/μL)	NA	385 (329–656)			
CD4 T cell count (cells/μL)	ND	452 (382–711)	580 (507–752)*	605 (480–942)*	741 (588–1037)*
ΔCD4 T cell count (cells/μL)	NA		135 (16–203)	129 (−22–209)	205 (53–462)
Viral load (RNA copies/mL)^c^	NA	41,050 (17,219–148,606)	20 (20–44)***	20 (20–20)***	20 (20–20)**
Undetectable viral load, *n* (%)	NA	0	13 (65%)	19 (95%)	10 (100%)

*NA, not applicable; ND, not determined*.

*^a^Longitudinal analysis was performed on HIV^+^ MSM only*.

*^b^Not statistically different to HIV^−^ MSM as determined by Chi-squared test*.

*^c^Values of <20 copies/mL were designated as 20 for the purpose of statistical analysis*.

*p < 0.05, **p < 0.01, ***p < 0.001 vs baseline from Wilcoxon matched pairs signed rank tests.

### Viremic HIV Infection Is Associated with Increased NK Cell Activation and Elevated Levels of HCMV Antibodies

Given our findings regarding the influence of MSM-related factors on FcRγ^−^ NK cell expansion, it was important to confirm the effect of HIV on NK cell activation and other immune parameters in an adequately controlled cohort. Compared to HIV^−^ MSM, viremic, cART-naive HIV^+^ MSM had significantly increased levels of NK cell activation as indicated by proportions of either HLA-DR^+^/CD38^+^ (median [IQR] 4.5% [2.5–5.9%] in HIV^+^ MSM vs 2.3% [1.4–3.0%] for HIV^−^ MSM, *p* = 0.015) or CD69^+^ NK cells (7.4% [4.5–10.8%] vs 3.2% [2.2–6.2%], *p* = 0.004) (Figures 2B,C, respectively). In addition to the increase in HCMV antibody levels associated with MSM status shown in Figure 1, viremic HIV infection was associated with a further increase in antibody levels to both whole HCMV lysate (*p* = 0.046, Figure 2D) and the gB antigen (*p* = 0.011, Figure 2E), while antibody levels to HCMV IE-1 did not differ with HIV status (data not shown).

Analysis of T cell and monocyte activation markers confirmed the well-established effect of viremic HIV infection on heightened CD4^+^ and CD8^+^ T cell activation (as assessed by HLA-DR/CD38 coexpression; *p* < 0.0001 for both vs HIV^−^ MSM, Figures 2F,G), expansion of inflammatory intermediate CD14^++^CD16^+^ monocytes (*p* < 0.0001 vs HIV^−^ MSM, Figure 2H), and concomitant reduction in classical CD14^++^CD16^−^ monocyte proportion (*p* = 0.008, data not shown). Taken together, data from this unique MSM cohort confirm that untreated HIV infection is associated with increased NK cell activation and generalized adaptive and innate immune activation, and indicate that this occurs in addition to the effects related to MSM status shown in Figure 1. Although HCMV seropositivity was near-ubiquitous in this cohort and not significantly different between HIV^+^ and HIV^−^ MSM (Table 1), a sub-analysis of only HCMV^+^ MSM indicated the proportion of activated HLA-DR^+^/CD38^+^, CD69^+^, and FcRγ^−^ NK cells was significantly higher in HIV^+^ vs HIV^−^ MSM (*p* = 0.032, 0.012, and 0.001, respectively, data not shown), indicating an effect of HIV independent of HCMV serostatus.

### Viral Suppression Associated with cART Does Not Reverse NK Cell Activation or the Expansion of FcRγ^−^ NK Cells

We undertook longitudinal analyses of pre- and post-cART samples from HIV^+^ MSM to determine the extent to which cART was able to reverse HIV-associated defects to NK cells as compared to other cellular/immunological compartments. Sixty-five percent, 95%, and 100% of individuals achieved undetectable viral load (<20 copies/mL) after 6, 12, and 24 months of cART, respectively (Table 1), and significant increases in CD4^+^ T cell counts observed at all post-cART time-points (*p* < 0.05 for all vs baseline, Table 1) confirmed the efficacy of cART in this cohort. The relatively high nadir CD4^+^ T cell count of 385 [329–656] (median [IQR]) and baseline CD4^+^ T cell count of 452 [382–711] cells/μL are typical of a contemporary HIV cohort where individuals initiate cART at higher CD4^+^ T cell counts prior to experiencing significant immunological damage.

Descriptive statistical analysis revealed that cART had no impact on proportions of FcRγ^−^ NK cells, which remained similar to pre-cART levels at 6, 12, and 24 months post-cART initiation (Figure 2A). Levels of activated HLA-DR^+^/CD38^+^ NK cells were also unaltered by cART and remained similar to levels in viremic, cART-naïve individuals at all follow-up time-points (Figure 2B). However, the proportion of NK cells expressing the early activation marker CD69 at 6, 12, and 24 months post-cART time-points were significantly lower than pre-cART levels (*p* = 0.004, 0.036, and 0.008, respectively; Figure 2C). Viral suppression associated with cART did not alter levels of HCMV antibodies to either whole lysate or gB antigens (Figures 2D,E, respectively). The lack of an effect of cART on NK cell dysfunction and HCMV antibody levels was in contrast to its effect on T cells and monocytes, where HIV-related CD8^+^ T cell activation and intermediate monocyte expansion were reversed to levels observed in uninfected MSM within 6 months of cART initiation (Figures 2G,H, respectively), while CD4^+^ T cell activation was resolved within 24 months of cART (Figure 2F). These descriptive analyses indicate cART is less effective at decreasing activation of NK cells compared to T cells and monocytes and demonstrate the persistence of NK cell dysfunction and elevated HCMV antibodies in HIV^+^ individuals despite 24 months of viral suppression.

### cART Reverses HIV-Related Activation of T Cells and Monocytes More Rapidly Than NK Cell Activation

To fully and quantitatively model the differential effect of cART on individual immune cell types indicated by the above descriptive analyses, we employed a mixed effects modeling framework to compare the rate of decline of HIV-related NK cell activation with other cellular compartments. This analytical approach has the additional benefit of accounting for the inherent variation in each individual’s initial immunological status and the way they subsequently respond to therapy, permitting a more accurate modeling of the change to specific immunological parameters over time in response to cART than is achievable with simple descriptive statistical analyses.

Latent growth-curve modeling confirmed cART had no significant effect on proportions of activated HLA-DR^+^/CD38^+^ NK cells [Wald χ^2^(1) = 2.3, *p* = 0.129] or FcRγ^−^ NK cells [Wald χ^2^(1) = 2.5, *p* = 0.115], while the HIV-related increase in activated CD69^+^ NK cell proportion declined significantly over time on cART [Wald χ^2^(1) = 9.1, *p* = 0.003, Table 2]. Our modeling also confirmed that T cell activation [Wald χ^2^(2) = 32.5 and Wald χ^2^(2) = 68.1 for CD4^+^ and CD8^+^ T cells, respectively, *p* < 0.001 for both, Table 2] and inflammatory intermediate monocyte subset expansion [Wald χ^2^(2) = 61.2, *p* < 0.001] were significantly reduced over time on cART.

**Table 2:** Latent growth-curve modeling^a^ showing associations between immune parameter outcomes and time (linear and quadratic) post-combination antiretroviral therapy (post-cART) initiation in HIV^+^ individuals (*n* = 20).

Immune parameter outcome	*b* (SE)	95% CI	Wald χ^2^	*p*-Value
**Activated/adaptive-like NK cells**				
NK cell (% CD69^+^)			χ^2^(1) = 9.1	0.003
Linear	−0.12 (0.04)	−0.21; −0.04		
Quadratic	–	–	–	–
NK cell (% HLA DR^+^/CD38^+^)	–	–	χ^2^(1) = 2.3^c^	0.129
Linear	−0.05 (0.03)	−0.11; 0.01	–	–
Quadratic	–	–	–	–
CD56^dim^ FcRγ^−^ NK cells			χ^2^(1) = 2.5	0.115
Linear	−0.22 (0.14)	−0.50; 0.05	–	–
Quadratic	–	–	–	–
**T cell activation**				
CD4^+^ T cell (% HLA DR^+^/CD38^+^)	–	–	χ^2^(2) = 32.5^b^	<0.001
Linear	−0.23 (0.05)	−0.34; −0.13	–	–
Quadratic	0.004 (0.002)	0.0004; 0.01	–	–
CD8^+^ T cell (% HLA DR^+^/CD38^+^)	–	–	χ^2^(2) = 68.1	<0.001
Linear	−1.08 (0.16)	−1.38; −0.77	–	–
Quadratic	0.03 (0.01)	0.01; 0.04	–	–
**Monocyte subsets**				
% Classical monocytes			χ^2^(2) = 28.5	<0.001
Linear	1.49 (0.39)	0.72; 2.26	–	–
Quadratic	−0.07 (0.03)	−0.13; −0.01		
% Intermediate monocytes			χ^2^(2) = 61.2	<0.001
Linear	−0.78 (0.11)	−1.00; −0.56	–	–
Quadratic	0.03 (0.01)	0.02; 0.05	–	–
% Non-classical monocytes			χ^2^(1) = 10.9	0.001
Linear	−0.30 (0.09)	−0.48; −0.12	–	–
Quadratic	–	–	–	–

*Table shows regression coefficient (*b*), SE, 95% confidence intervals (95% CI), Wald tests (Wald χ^2^) and probability value (*p*-value)*.

*^a^Latent growth-curve modeling specifying a random intercept (baseline marker level) and coefficient (linear time) and unstructured covariance terms for random effects*.

*^b^Intercept and slope covariance term not able to be computed for this model*.

*^c^Random intercept model only—random coefficient model did not converge*.

*Note: Where a quadratic coefficient is not shown for an outcome, nested likelihood ratio tests did not reject the null hypothesis that the functional form of time on cART was linear*.

To compare the rate at which cART reversed CD69^+^ NK cell, T cell and monocyte activation, the proportion of pre-cART immune activation, which remained after 6 and 12 months on cART, was calculated for each cell type (Table 3). After 12 months of cART, 60% (95% CI:53–67%) of activated CD4^+^ and 30% (21–39%) of activated CD8^+^ T cells remained, indicating a respective 40 and 70% reduction in T cell activation. Similarly, 12 months of cART was associated with a 53% (44–62%) and 85% (54–138%) reversal of the HIV-related alterations to intermediate and classical monocyte subset proportions, respectively. In contrast, reversal of activated CD69^+^ NK cells was substantially slower, with only 20% (13–27%) of HIV-related activation reversed following 12 months of cART.

**Table 3:** Exponentiated regression coefficient indicating percent change in log immunological parameters from baseline after 6 and 12 months of combination antiretroviral therapy from latent growth-curve modeling (*n* = 20).

	6 months	12 months
Immune parameter outcome	Exp *b* (95% CI)^a^	Exp *b* (95% CI)^a^
**NK cell activation/function**		
NK cell (% CD69^+^)	**0.89 (0.85,0.93)**	**0.80 (0.73,0.87)**
NK cell (% HLA DR^+^/CD38^+^)	0.94 (0.88,1.01)	0.89 (0.76,1.02)
CD56^dim^ FcRγ^−^ NK cells	0.94 (0.88,0.99)	0.88 (0.77,0.99)
**T cell activation**		
CD4^+^ (% HLA DR^+^/CD38^+^)	**0.77 (0.73,0.82)**	**0.60 (0.53,0.67)**
CD8^+^ (% HLA DR^+^/CD38^+^)	**0.50 (0.41,0.60)**	**0.30 (0.21,0.39)**
**Monocyte subsets**		
% Classical monocytes	**1.36 (1.23,1.51)**	**1.85 (1.54,2.38)**
% Intermediate monocytes	**0.56 (0.46,0.66)**	**0.47 (0.38,0.56)**
% Non-classical monocytes	**0.74 (0.64,0.84)**	**0.55 (0.40,0.70)**

*^a^Exponentiated regression coefficients (Exp *b*) and 95% confidence intervals (95% CI) from non-linear combined (i.e., linear and quadratic terms) effect estimation based on log-normal latent growth-curve models—coefficient (Exp *b*) represents the ratio of expected geometric mean difference in a marker for a specific length of time taking account of the functional form of the effect of time (i.e., % change in a marker per specified time-period). Ratios <1 indicate a percent decrease. Values in **bold** indicate marker levels exhibiting significant change over-time*.

**Figure 3:**
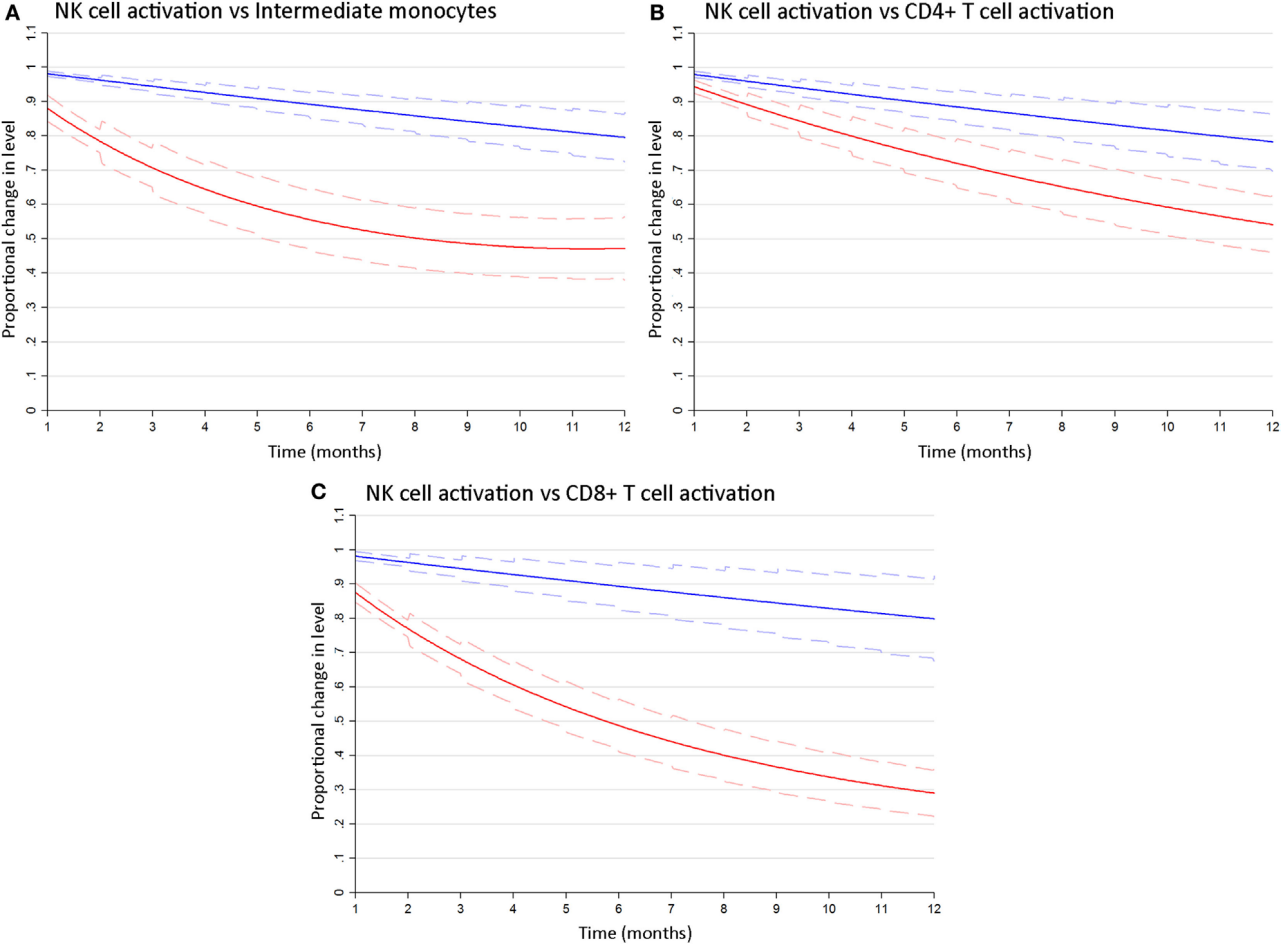
NK cell activation decays more slowly following combination antiretroviral therapy (cART) initiation than T cell or monocyte activation. Bivariate latent growth curve models comparing the modeled rate of decline in the proportion of activated CD69^+^ NK cells (blue lines) vs inflammatory intermediate CD14^++^CD16^+^ monocytes [red lines, **(A)**], activated HLA-DR^+^/CD38^+^ CD4^+^ [red lines, **(B)**] or CD8^+^ [red lines, **(C)**] T cells for 12 months after cART initiation. Mean exponentiated effects and 95% confidence intervals are shown (solid and dashed lines, respectively). Note: Plots show mean exponentiated linear/quadratic effects by time after cART initiation and 95% confidence intervals. Values <1 indicate a reduction in marker level over time.

To determine whether differences in the rates at which cART reversed HIV-related activation of NK cells as compared to T cells and monocytes were significant, we estimated bivariate latent-growth curve models regressing each of the marker outcomes on respective functions of time simultaneously and allowing each individual’s treatment responses in marker levels to correlate. This revealed significant differences in the rate of reversal of CD69^+^ NK cell activation as compared to inflammatory intermediate monocyte expansion [6 months: Wald χ^2^(1) = 42.6; 12 months: Wald χ^2^(1) = 28.0; *p* < 0.001 for both, Figure 3A]. Similarly, the proportion of activated CD69^+^ NK cells declined significantly more slowly after cART initiation than activated (HLA-DR^+^/CD38^+^) CD4^+^ T-cells [6 months: Wald χ^2^(1) = 13.7; 12 months: Wald χ^2^(1) = 16.1, *p* < 0.001 for both, Figure 3B] or CD8^+^ T-cells [6 months: Wald χ^2^(1) = 58.3; 12 months Wald χ^2^(1) = 47.8, *p* < 0.001 for both, Figure 3C]. These analyses indicate that cART has a differential effect on different arms of the immune system, and robustly confirm that NK cell activation resolves more slowly than T cell or monocyte dysfunction following cART initiation.

### Expansion of Adaptive FcRγ^−^ NK Cells Is Not Associated with HCMV Antibody Levels or Other Immune Activation Markers in HIV^+^ Individuals

Given the persistence of both FcRγ^−^ NK cells and elevated HCMV antibody levels in cART-experienced HIV^+^ MSM, and our previous observation of an association between these two parameters in HIV-uninfected individuals (3), we extended the modeling to investigate associations between FcRγ^−^ NK cells and elevated HCMV antibody levels in HIV^+^ MSM. Contemporaneous time-varying associations between individuals’ FcRγ^−^ NK cell levels and other key markers were estimated from longitudinal data obtained up to 12 months post-cART initiation using linear mixed modeling, which permitted the analysis of repeated measures data from the same individuals. This analysis indicated that FcRγ^−^ NK cell levels were not associated with levels of antibody to either HCMV lysate [Wald χ^2^(1) = 0.23, *p* = 0.631] or gB [Wald χ^2^(1) = 0.89, *p* = 0.345, Table 4]. Significantly, there was no association between the proportions of FcRγ^−^ NK cells and NK cell activation (either HLA-DR^+^/CD38^+^ or CD69^+^ NK cells, *p* = 0.705 and 0.182, respectively, Table 4), nor with any other cellular or soluble immune activation marker measured. This lack of association was also observed when only baseline samples from cART naïve individuals were analyzed (data not shown). These findings are consistent with our previous cross-sectional study that indicated an association between HCMV antibody levels and adaptive-like FcRγ^−^ NK cells in HIV^−^ but not HIV^+^ individuals (3).

**Table 4:** Mixed modeling^a^ showing unadjusted longitudinal associations between FcRγ^−^ CD56^dim^ FcRγ^−^ NK cell levels and immune parameters in HIV^+^ individuals (*n* = 20).

Immune parameter	*b*(SE)	95% CI	Wald χ^2^	*p*-Value
**T cell activation**				
CD4^+^ (% HLA DR^+^/CD38^+^)	0.33 (0.66)	−0.96, 1.62	χ^2^(1) = 0.26	0.613
CD8^+^ (% HLA DR^+^/CD38^+^)	0.01 (0.21)	−0.40, 0.41	χ^2^(1) = 0.00	0.977
**NK cell activation/function**				
NK (% CD69^+^)	0.62 (0.46)	−0.29,1.52	χ^2^(1) = 1.78	0.182
NK (% HLA DR^+^/CD38^+^)	−0.24 (0.63)	−1.46, 0.99	χ^2^(1) = 0.14	0.705
**Monocyte subsets**				
% Classical monocytes	−0.13 (0.20)	−0.51, 0.26	χ^2^(1) = 0.42	0.519
% Intermediate monocytes	0.35 (0.46)	−0.55, 1.25	χ^2^(1) = 0.59	0.444
% Non-classical monocytes	0.14 (0.30)	−0.44, 0.72	χ^2^(1) = 0.23	0.631
**Soluble markers**				
HCMV lysate IgG (AU)	2.4 × 10^−5^ (4.9 × 10^−5^)	−7.3 × 10^−5^, 1.2 × 10^−4^	χ^2^(1) = 0.23	0.631
HCMV gB IgG (AU)	2.7 × 10^−5^ (2.9 × 10^−5^)	−2.9 × 10^−5^, 8.4 × 10^−5^	χ^2^(1) = 0.89	0.345
CXCL10 (pg/mL)	2.7 × 10^−3^ (0.01)	−0.02, 0.03	χ^2^(1) = 0.05	0.828
sCD163 (ng/mL)	3.5 × 10^−3^ (2.2 × 10^−3^)	−8.7 × 10^−4^, 7.9 × 10^−3^	χ^2^(1) = 2.47	0.116

*Regression coefficient (*b*), SE, 95% confidence intervals (95% CI), Wald test (Wald χ^2^), and probability value (*p*-value)*.

*^a^Linear mixed modeling specifying a random intercept for study participant to account for the dependency associated with repeated measurements (i.e., all participant observations were used in analyses)*.

*AU, arbitrary units*.

In contrast to levels of FcRγ^−^ NK cells, NK cell activation, measured as either HLA-DR^+^/CD38^+^ or CD69^+^ NK cells, was significantly associated with CD4^+^ T cell activation [Wald χ^2^(1) = 5.31, *p* = 0.021 and Wald χ^2^(1) = 9.55, *p* = 0.002, respectively] and the proportion of intermediate [Wald χ^2^(1) = 4.29, *p* = 0.038 and Wald χ^2^(1) = 6.02, *p* = 0.014, respectively] and classical [Wald χ^2^(1) = 5.71, *p* = 0.017 and Wald χ^2^(1) = 4.36, *p* = 0.037, respectively] monocyte subsets (Table S1 in Supplementary Material). Activated CD69^+^ NK cells were also significantly associated with CD8^+^ T cell activation [Wald χ^2^(1) = 9.24, *p* = 0.002] and plasma levels of CXCL10 [Wald χ^2^(1) = 6.66, *p* = 0.010], whilst neither HLA-DR^+^/CD38^+^ or CD69^+^ activated NK cells were associated with HCMV antibody levels (*p* > 0.05 for all). These findings suggest that whilst NK cell activation is associated with other markers of adaptive and innate immune activation in HIV^+^ individuals, the expansion of adaptive-like FcRγ^−^ NK cells is a discrete phenomenon.

## Discussion

HIV infection has a profound impact on NK cells including heightened cellular activation, imprinting of the NK cell receptor repertoire and expansion of NK cell subpopulations with an adaptive, memory-like phenotype (2, 3, 26–28). Studies investigating the impact of HIV infection and cART on immunological parameters are often limited by cross-sectional study designs and inappropriate HIV^−^ comparator populations that do not adequately control for confounders such as heightened HCMV seropositivity. In this study, we used a unique and carefully controlled longitudinal cohort of HIV^+^ MSM initiating cART with HIV-seronegative MSM recruited from the same primary care sites to investigate the impact of HIV infection and cART on NK cell dysfunction. We found the proportion of CD56^dim^ FcRγ^−^ NK cells was significantly increased in cART-naïve HIV^+^ MSM as compared to HIV^−^ MSM, who in turn were immunologically distinct from community controls. Viremic HIV infection in MSM was also associated with significant activation of CD56^dim^ NK cells, as assessed using HLA-DR/CD38 co-expression as described previously (2) or CD69 expression as phenotypic markers of activation. CD69 ligation induces cytolytic activity in NK cells (36), but it is also involved in retention of lymphocytes in lymphoid tissue (37) and is highly expressed on tissue-resident NK cells (38), suggesting HIV infection may be associated with greater trafficking of NK cells between blood and tissues. Expression of the maturation marker CD57 on CD56^dim^ NK cells was also unaltered by cART in this cohort (data not shown), which is consistent with our previous findings (31).

To robustly quantify the effect of virologic suppression on NK cell dysfunction, we used mixed effects modeling to account for inherent differences in each individuals’ immunological response to HIV infection and subsequent therapy. Importantly, unlike T lymphocyte and monocyte activation, which declined significantly following cART initiation, the expansion of FcRγ^−^ NK cells in HIV^+^ MSM was not affected by cART and appeared to represent a stable population present in the setting of HIV infection. Similarly, Brunetta et al. found that proportions of NKG2C^+^ NK cells (a population analogous to the FcRγ^−^ NK cells described here (3)) remained consistently elevated despite 2 years of cART, while HIV-associated changes to the ratio of NKG2A^+^/NKG2C^+^ NK cells were normalized during this period (27). Together, these data indicate an enduring effect of HIV infection on NK cell dysfunction in virologically suppressed HIV^+^ individuals, which persists for at least 2 years following cART initiation and long after normalization of other immune parameters.

The persistence of activated HLA-DR^+^/CD38^+^ NK cells in cART-treated HIV^+^ MSM demonstrated by our latent growth curve modeling unequivocally confirms observations from our cross-sectional studies, which found heightened NK cell activation (measured using phenotypic markers or spontaneous degranulation) in virologically suppressed HIV^+^ individuals (2, 3). This implies NK cell activation is driven by factors other than HIV viremia, although it is still possible that NK cells are more sensitive to residual viral replication (<20 RNA copies/mL detected using validated clinical assays) than other leukocyte types. The association between NK cell and monocyte activation observed here suggests monocyte activation, potentially resulting from endotoxemia, may contribute to NK cell activation in viremic HIV infection. However, the persistence of NK cell activation but not monocyte activation in cART-treated individuals implies either that other factors maintain NK cell activation in virologically suppressed individuals or that NK cells are more sensitive indicators of persistent immune dysfunction. Interestingly, we did not observe an association between NK cell activation and HCMV antibody levels, although HCMV antibody levels alone are an imperfect metric of an individual’s HCMV burden and the frequency and magnitude of reactivation events, particularly in HIV infection, as antibody levels in individuals with advanced HIV disease can initially rise following cART-initiation, and then subsequently fall (39, 40). Quantitation of the burden of HCMV remains problematic as the virus replicates in tissue cells, so viral DNA may not be detectable in blood. Thus, it remains possible that HCMV replication may contribute to persistent NK cell activation in cART-treated HIV infection.

In contrast to HLA-DR^+^/CD38^+^ NK cells, there was a slow but significant decrease in CD69^+^ NK cells following cART initiation, consistent with CD69 being an early marker of cellular activation known to decline during the convalescent stage of viral infections such as Hantavirus (41). However, the rate at which activated CD69^+^ NK cells declined on cART was significantly slower than that observed for T lymphocyte and monocyte activation, indicating NK cells are a cell population very sensitive to inflammatory or immune stimulatory factors that remain after antiretroviral therapy. Monitoring NK activation may therefore be a robust indicator of residual immune dysfunction in cART-treated individuals and could also be a useful biomarker for monitoring the efficacy of future functional cure strategies aimed at supressing HIV viremia and related immune activation in the absence of cART.

In HIV-uninfected individuals, FcRγ^−^ NK cells are generated in response to HCMV infection (22, 42). We have reported that the frequency of these cells correlates strongly with HCMV antibody levels in HIV^−^ individuals recruited from the community (3), suggesting that in the general population, levels of HCMV infection and/or reactivation (to the extent indicated by antibody responses) are driving the expansion of NK cells with adaptive immune properties. In contrast, we have shown both here and previously (3) that HCMV antibody levels do not correlate with FcRγ^−^ NK cell frequencies in HIV^+^ MSM. It remains possible that HCMV infection is a prerequisite for the HIV-associated expansion of FcRγ^−^ NK cells observed here, similar to the effect of HIV on NKG2C^+^ NK cell expansion, which is only observed in HCMV^+^ individuals (27). Furthermore, HCMV reactivation may in fact be a primary driver of FcRγ^−^ NK cell expansion in HIV infection, but the abovementioned limitations with HCMV antibody levels as indicators of HCMV burden may preclude detection of this relationship. We have previously shown the phenotype of adaptive-like NK cells expanded in response to HCMV infection in renal transplant patients differs from those expanded in HIV infection (31, 43), implying that HIV-related factors may drive expansion of adaptive-like NK cells in addition to the effects of HCMV. The lack of an association between FcRγ^−^ NK cells and immune activation found in this study suggests adaptive-like NK cell expansion is driven by mechanisms distinct from immune cell activation.

Adaptive-like NK populations are characterized phenotypically by reduced expression of the inhibitory receptor NKG2A and signaling molecules such as FcRγ, Syk, and Siglec-7 and increased expression of the activating receptor NKG2C (23, 32). FcRγ^−^ NK cells lack the cytotoxic receptors NKp30 and NKp46 (3) and have poor cytotoxic activity against tumor targets (32); thus, the increased proportion of these NK cells may contribute to the increased prevalence of non-AIDS malignancies seen in HIV^+^ individuals, although this requires formal investigation. As NKp46 is required for killing of HIV-infected CD4^+^ T cells by natural cytotoxicity (44), the accumulation of NKp46^−^ FcRγ^−^ NK cells in HIV^+^ individuals may impair NK-mediated clearance of HIV-infected T cells, although this may be counteracted by their enhanced ADCC activity. The enhanced ability of FcRγ^−^ NK cells to produce inflammatory factors such as TNF and IFNγ following antibody stimulation (22, 32) may also perpetuate inflammation and immune activation in cART-treated individuals. Given that adaptive-like NK cells are expanded and persist in response to chronic viral infection it is reasonable to speculate that they play a role in protective immunity, although their contribution to this process *in vivo* remains to be determined. Consistent with this, NKG2C^bright^ (45) and FcRγ^−^ (22) NK cells expanded in HCMV^+^ individuals show heightened antibody-mediated degranulation, cytokine production, and ADCC against not only HCMV but also HSV-1 targets, implying a role in antibody-dependent cross-protection. However, HIV^+^/HCMV^+^ individuals have higher levels of HCMV antibodies than individuals infected with HCMV alone (31), implying poor HCMV control. It is plausible that abundant antibody and FcRγ^−^ NK cells together compensate for poor protective T-cell responses in HIV^+^ individuals. We found FcRγ^−^ NK cells isolated from HIV^+^ individuals have increased *ex vivo* ADCC activity when stimulated by HIV peptides in the presence of heterologous HIV^+^ serum (3), but whether this translates to enhanced killing of HIV-infected cells *ex vivo* or *in vivo*, and whether this affects HIV reservoirs, is an important question that warrants investigation.

This study presents unique longitudinal data examining HIV-related immune activation specifically in MSM by comparison to matched HIV^−^ MSM controls. The concentration of the HIV epidemic in MSM populations in many developed countries including Australia (46) means that MSM are overrepresented in clinical HIV studies conducted in these settings, but MSM-related factors are rarely considered as potential confounders. Our finding of increased proportions of FcRγ^−^ NK cells and elevated HCMV antibody levels in HIV-uninfected MSM as compared to community controls underscores the importance of using appropriately matched, MSM controls to study immunological changes in HIV^+^ MSM.

This study has a number of limitations, including a relatively small sample size, although this cohort size was chosen since, with 20 participants, the study provides a minimum number of level-two units to reliably estimate fixed model parameters in longitudinal mixed modeling (47–49). Other limitations include the absence of female participants, the use of an exclusively MSM cohort, and a follow-up of only 2 years. Follow-up of the cohort is ongoing and future analysis of later post-cART time-points will be critical for determining whether periods of cART >2 years are able to mitigate FcRγ^−^ NK cell expansion. This study has however highlighted a significant and enduring effect of chronic, virologically suppressed HIV infection on the activation and imprinting of NK cells. Identification of the mechanisms responsible for the creation and maintenance of the expanded adaptive-like NK cell population in HIV^+^ individuals, and the clinical consequences of their expansion, will inform adjunct immunotherapies to adequately address persistent immune dysfunction in cART-treated HIV infection.

## Ethics Statement

This study was approved by the Alfred Hospital Research and Ethics Committee and carried out in accordance with their recommendations. All subjects gave written informed consent in accordance with the Declaration of Helsinki.

## Author Contributions

AH, JZ, SB, MC, and TA generated experimental data; AH, PA, MG, PC, PP, JE, and AJ contributed to study design and interpretation of the data; and AH, PA, and AJ analyzed the data and prepared the manuscript (with approval from all authors).

## Conflict of Interest Statement

The authors declare that the research was conducted in the absence of any commercial or financial relationships that could be construed as a potential conflict of interest.
