# Supplementary material for: Persistence of Activated and Adaptive-Like NK Cells in HIV+ Individuals despite 2 Years of Suppressive Combination Antiretroviral Therapy
Source: Front Immunol. 2017 Jun 30;8:731. doi: 10.3389/fimmu.2017.00731 (PMC5491541; doi:10.3389/fimmu.2017.00731)
Supplement: Supplementary file 1 [file Data_Sheet_1.PDF]

Supplementary Figure 1

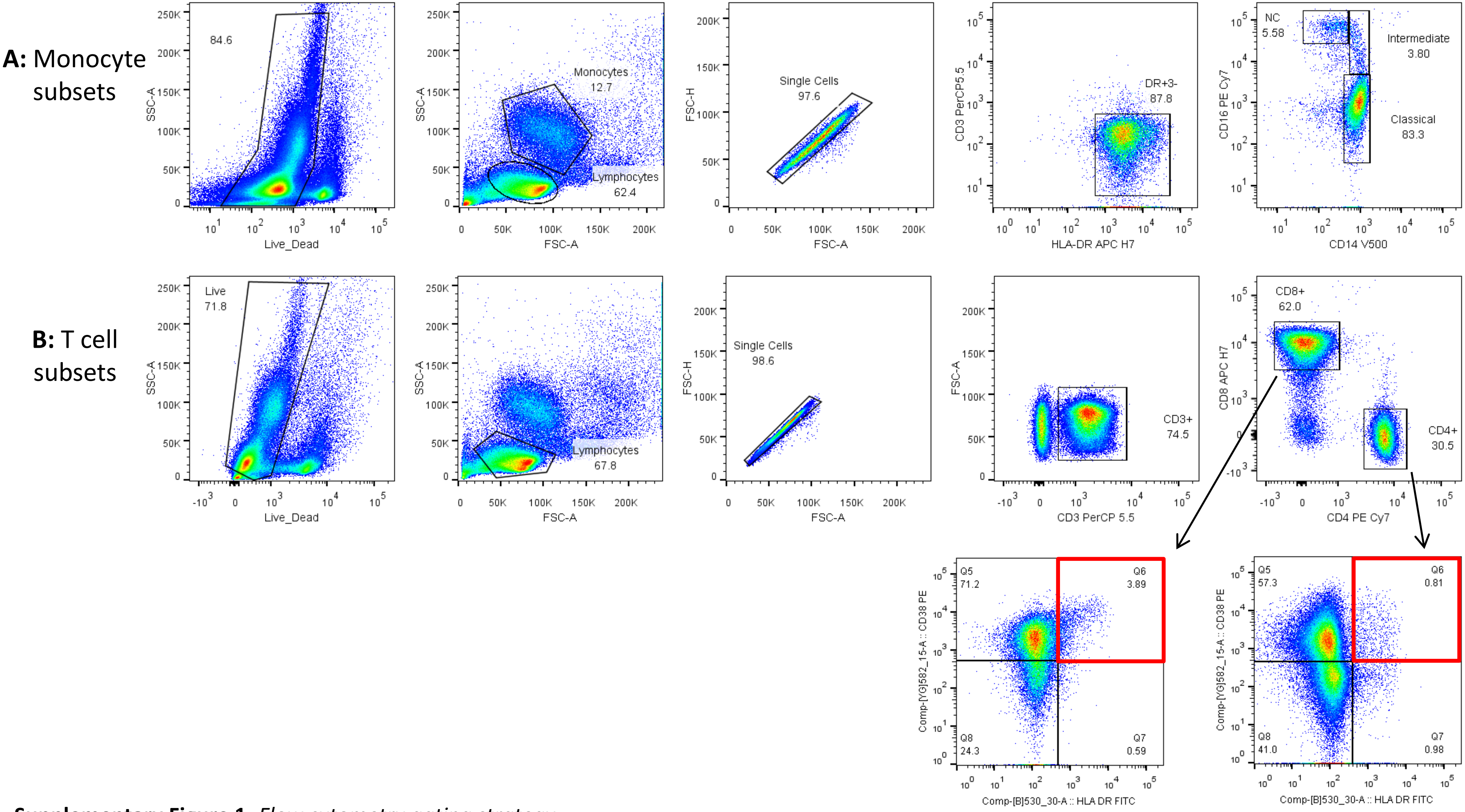

Supplementary Figure 1: *Flow cytometry gating strategy.*

Gating of all cell populations involved exclusion of non-viable cells positive for the LIVE/DEAD® marker, subsequent gating of the relevant monocyte/lymphocyte population via forward vs side scatter (area) and gating of single cells via forward scatter area vs height. A minimum of 400 cells per subset were anlaysed. **A:** Monocytes were gated as $CD3^{-}HLA\text{-}DR^{+}$ cells then into Classical ($CD14^{++}CD16^{-}$), Intermediate ($CD14^{++}CD16^{+}$) and Non-classical ($CD14^{+}CD16^{++}$) subsets. **B:** T cells were gated as $CD3^{+}$ and either $CD4^{+}$ or $CD8^{+}$ subsets, then as $CD38^{+}$/$HLA\text{-}DR^{+}$ as shown. **C:** $CD56^{dim}$ NK cells were gated as $CD3^{-}CD56^{dim}CD16^{+}$ cells, and as FcRγ-, $CD38^{+}$/$HLA\text{-}DR^{+}$ or $CD69^{+}$ as shown (overpage).

Supplementary Figure 1 (cont.)

C:
CD16^{+}CD56^{dim}
NK cells

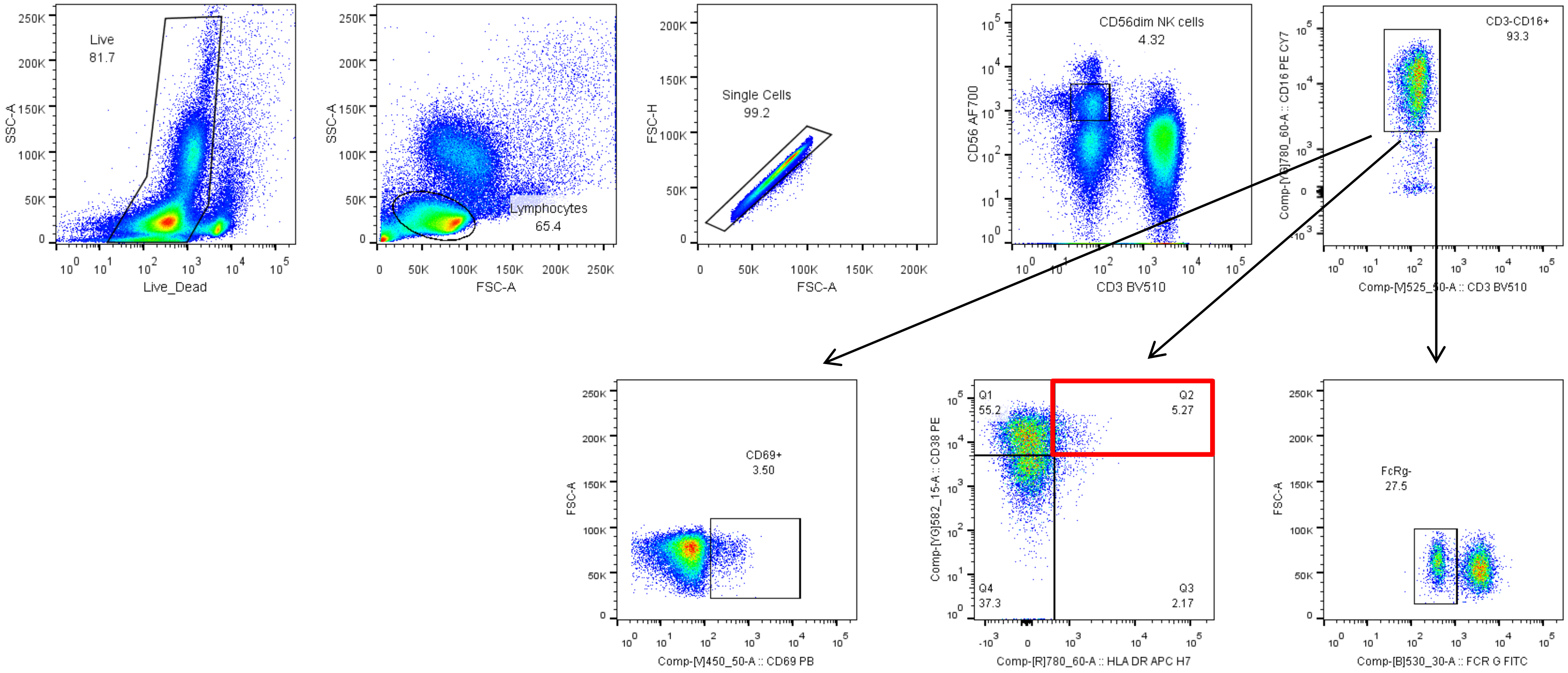

CD69+ **CD38+/HLA-DR+** **FcRγ-**

Supplementary Figure 2

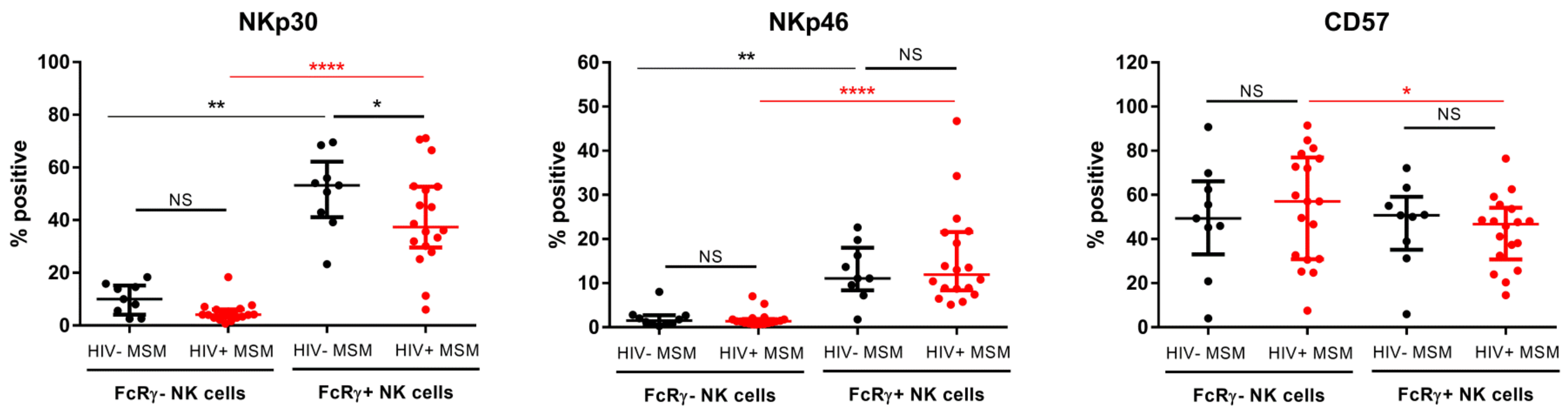

Supplementary Figure 2: *The phenotype of adaptive CD56^{dim} FcRγ- NK cells is similar in both HIV+ and uninfected MSM.*
The expression of NKp30 (A), NKp46 (B) and CD57 (C) on FcRγ^{-} and FcRγ^{+} CD56^{dim} NK cells from both HIV+ MSM (n=19) and uninfected MSM controls (n=10) was determined by immunophenotyping. Only individuals with >5% FcRγ^{-} NK cells were included in this analysis. Graphs show paired results for FcRγ^{-} and FcRγ^{+} NK cells from each individual. *, ** and *** denote p<0.05, 0.01 and 0.001 respectively compared to uninfected MSM controls as determined by Wilcoxon matched-pairs signed rank test.

$$y_{ij} = \beta_1 + \beta_2 t_{ij} + \beta_3 t^2{}_{ij} + \zeta_{1j} + \zeta_{2j} t_{ij} + \epsilon_{ij} \quad \text{with } \zeta_{nj} \sim N(0,\sigma^2)$$

Where β_1 is the mean baseline marker level, $(\beta_2 t_{ij} + \beta_3 t^2{}_{ij})$ the linear and squared terms representing the functional form of time post treatment for person *j* at occasion *i*, ζ_{1j} the random effect (i.e. intercept) for between-person variability in baseline marker level , $\zeta_{2j} t_{ij}$ the random effect (i.e. coefficient) for between-person variability in marker level change across time and ϵ_{ij} the person-specific between-response residual.

Supplementary Equation 1: *Latent-growth curve model equation*
The equation for the latent growth-curve model used to investigate the association between immunological markers and time post-cART initiation in HIV+ individuals.

Supplementary Table 1: Mixed modelling† showing unadjusted longitudinal associations between activated HLA-DR+/CD38+ or CD69+NK cell proportions and immune parameters in HIV+ individuals.

	**HLA-DR+/CD38+ NK cells**				**CD69+ NK cells**			
**Immune parameter**	***b*(SE)**	**95% CI**	**Wald χ^2**	**p-value**	***b*(SE)**	**95% CI**	**Wald χ^2**	**p-value**
T cell activation								
% HLA-DR$^+$/CD38$^+$ CD4$^+$ T cells	0.30 (0.13)	0.04, 0.55	5.31	**0.021**	0.46 (0.15)	0.17, 0.75	9.55	**0.002**
% HLA-DR$^+$/CD38$^+$ CD8$^+$ T cells	0.07 (0.04)	-0.02, 0.15	2.26	0.133	0.14 (0.05)	0.05, 0.23	9.24	**0.002**
Monocyte subsets								
% Classical	0.10 (0.04)	0.02, 0.18	5.71	**0.017**	-0.11 (0.05)	-0.21, -0.01	4.36	**0.037**
% Intermediate	-0.21 (0.10)	-0.42, -0.01	4.29	**0.038**	0.29 (0.12)	0.06, 0.53	6.02	**0.014**
% Non-classical	-0.14 (0.06)	-0.25, -0.02	4.96	**0.026**	0.13 (0.08)	-0.03, 0.29	2.46	0.117
Soluble markers								
HCMV lysate	-5.8×10^{-6} (6.1×10^{-6})	-1.8×10^{-5}, 6.4×10^{-6}	0.87	0.352	2.5×10^{-5} (1.4×10^{-5})	-2.4×10^{-6}, 5.2×10^{-5}	3.19	0.074
HCMV gB	1.7×10^{-6} (4.2×10^{-6})	-6.6×10^{-6}, 1.0×10^{-6}	0.16	0.693	1.3×10^{-5} (7.8×10^{-6})	-2.0×10^{-6}, 2.8×10^{-5}	2.89	0.089
CXCL10	8.6×10^{-5} (2.7×10^{-3})	-5.2×10^{-3}, 5.3×10^{-3}	0.00	0.974	7.4×10^{-3} (2.9×10^{-3})	1.8×10^{-3}, 0.01	6.66	**0.010**
sCD163	4.4×10^{-4} (4.1×10^{-4})	-3.7×10^{-4}, 1.2×10^{-3}	1.13	0.288	9.1×10^{-4} (5.6×10^{-4})	-1.9×10^{-4}, 2.0×10^{-3}	2.61	0.106

† Linear mixed modelling with a random intercept for study participant to account for the dependency associated with repeated measurements (i.e. all participant observations were used in analyses). Regression coefficient (*b*), standard error (SE), 95% confidence intervals (95% CI), Wald tests (Wald χ^2) and probability value (p-value) are shown. Statistically significant p values are shown in bold.
